# Vascular Endothelial Galectins in Leukocyte Trafficking

**DOI:** 10.3389/fimmu.2021.687711

**Published:** 2021-06-01

**Authors:** Abbey Lightfoot, Helen M. McGettrick, Asif J. Iqbal

**Affiliations:** ^1^ Institute of Cardiovascular Sciences, University of Birmingham, Birmingham, United Kingdom; ^2^ Institute of Inflammation and Ageing, University of Birmingham, Birmingham, United Kingdom

**Keywords:** galectins, leukocyte trafficking, glycan-binding protein, endothelial cell, vascular biology

## Abstract

Leukocyte recruitment to the site of injury is a crucial event in the regulation of an inflammatory response. Tight regulation of interactions between the endothelium and circulating leukocytes is necessary to ensure a protective response to injury does not result in inflammatory disease. Rising interest in the broad immunoregulatory roles displayed by members of the glycan-binding galectin family suggests that these proteins could be an attractive target for therapeutic intervention, since their expression is significantly altered in disease. The focus of this review is to summarize current knowledge on the role of galectins in leukocyte trafficking during inflammation and the clinical approaches being taken to target these interactions for treatment of inflammatory disease.

## Introduction

Glycans are one of the four major components that constitute cells, accompanied by nucleic acids, proteins and lipids. Recognition of specific glycan motifs by glycan-binding proteins (lectins) is crucial for facilitating highly sophisticated cross-communication between leukocytes in the bloodstream and endothelial cells (ECs) lining the blood vessels (1). Several key lectins (E-, L- and P-selectins and Cluster of differentiation [CD] 44) have already been identified as mediators of leukocyte recruitment and trafficking at the site of injury in the initial stages of the immune response (1). β-galactoside-binding galectins have emerged as an interesting family of glycan-binding proteins involved in the initiation and resolution stages of the inflammatory response (2, 3). Our understanding of the roles of endothelial-expressed galectins in the leukocyte trafficking cascade is relatively poor when compared to other known glycan-binding proteins involved in this process. As such, this review will provide an up-to-date overview on the role of endothelial derived galectins in leukocyte trafficking; the factors that regulate their expression and function; and discusses the therapeutic potential of targeting these interactions to treat immune-mediated chronic inflammatory disease (IMID).

Upon injury or infection, tissue resident immune cells release pro-inflammatory factors such as reactive oxygen species and proteases that partially degrade glycoprotein components of the glycocalyx; an EC-expressed matrix that serves to shield the vascular walls from direct exposure to blood components and flow (4, 5). Glycosaminoglycans (GAGs) in the reduced layer of glycocalyx bind and immobilize chemokines, accompanied by the activation of ECs and upregulation of surface expressed adhesion molecules (6). Hemodynamic forces facilitate the outward movement of leukocytes towards the venular endothelium in a process known as margination, enabling interactions between leukocytes and newly exposed molecules displayed on the endothelium (6). Tight regulatory mechanisms are required to ensure leukocyte recruitment occurs only when appropriate, as such ECs are able to detect and respond to environmental changes such as the hemodynamic forces of blood flow (7, 8). Continuous pulsatile and laminar flow activates mechanosensitive channels and suppresses nuclear-factor kappa-B (NF-κB) *via* the MEK5/ERK5 (mitogen-activated protein kinase 5-extracellular signal-regulated kinase 5) pathway. Consequently, this prevents initiation of the inflammatory response through the downregulation of adhesion molecules and thus reduced leukocyte recruitment to the endothelium (9).

**Figure 1:**
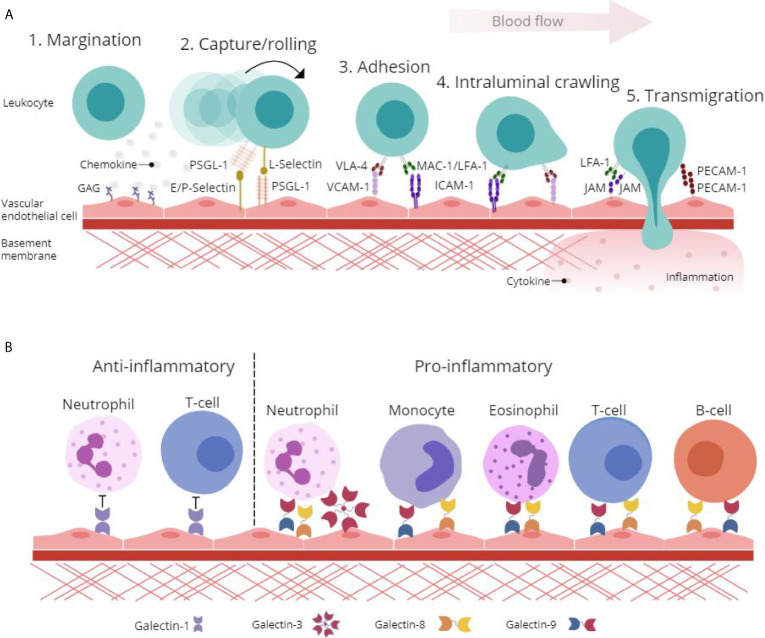
Glycan-binding proteins regulate endothelial-leukocyte interactions in inflammation. **(A)** Protein-protein and protein-glycan interactions are crucial regulators of leukocyte-endothelial interactions involved in the leukocyte adhesion cascade. Firstly, glycosaminoglycan (GAG)-presented chemokines promote leukocyte margination to the vessel wall. Capture and rolling of leukocytes along the endothelium is facilitated by interactions between selectins and their ligands such as P-selectin glycoprotein ligand (PSGL)-1. This interaction leads to cell activation and conformational changes in leukocyte integrins including Very Late Antigen (VLA)-4, Macrophage (Mac)-1 antigen and lymphocyte function-associated antigen (LFA)-1 that then interact with adhesion molecules such as Vascular Cell Adhesion Molecule (VCAM)-1 and Intracellular Cell Adhesion Molecule (ICAM)-1 to facilitate adhesion and intraluminal crawling. Finally, transmigration through the endothelial cell (EC) layer is mediated by homophilic interactions between platelet EC adhesion molecule (PECAM)-1 and junctional adhesion molecule (JAM). **(B)** β-galactoside binding galectins facilitate and inhibit leukocyte trafficking to the endothelium to promote pro- and anti-inflammatory responses. Galectin-1 inhibits leukocyte-endothelial interactions to promote an anti-inflammatory phenotype, whilst galectin-3, -8 and -9 facilitate pro-inflammatory leukocyte trafficking.

Adhesion molecules displayed on the surface of activated ECs, particularly selectins (E- and P-selectins), reduce the velocity of leukocytes rolling along the endothelium through binding to heavily glycosylated counter-receptors [PSGL-1, P-selectin glycoprotein ligand-1 (CD162)] involved in the capture and subsequent rolling events of the leukocyte adhesion cascade (**Figure 1A**) (10). This interaction induces conformational changes in integrins [MAC-1, Macrophage antigen-1 (CD11b/CD18); VLA-4, very late antigen-4 (CD49d/CD29); and LFA-1, lymphocyte function-associated antigen-1 (CD11a/CD18)] and facilitates firm adhesion of leukocytes to the endothelium by binding EC-expressed immunoglobulin superfamily adhesion molecules [ICAM-1, Intercellular Adhesion Molecule 1 (CD54) and VCAM-1, Vascular cell adhesion protein 1 (CD106)] (11). Additionally, the interaction between integrins and adhesion molecules facilitates the intraluminal crawling of leukocytes across ECs (12). Migration of leukocytes through the endothelium occurs either at endothelial junctions between ECs (paracellular) or through the body of the EC itself (transcellular). This migration of leukocytes into the surrounding tissue is facilitated by leukocyte-integrin binding to junctional adhesion molecules (JAMs) and PECAM-1 [CD31] expressed on the EC surface (**Figure 1A**) (12).

Whilst many aspects of the leukocyte trafficking cascade have been studied in detail, a lot still remains unknown. Specifically, the galectin family of glycan-binding proteins have recently come to light as functionally important immunoregulatory proteins with involvement in leukocyte activation, apoptosis and in mediating leukocyte adhesion and migration (**Figure 1B**) (13, 14). Enhancing our understanding of the regulators of galectin expression and their roles in mediating leukocyte trafficking could uncover novel mechanisms of inflammation and highlight distinctive opportunities for the treatment of IMIDs.

## Galectin Structure and Signaling

**Figure 2:**
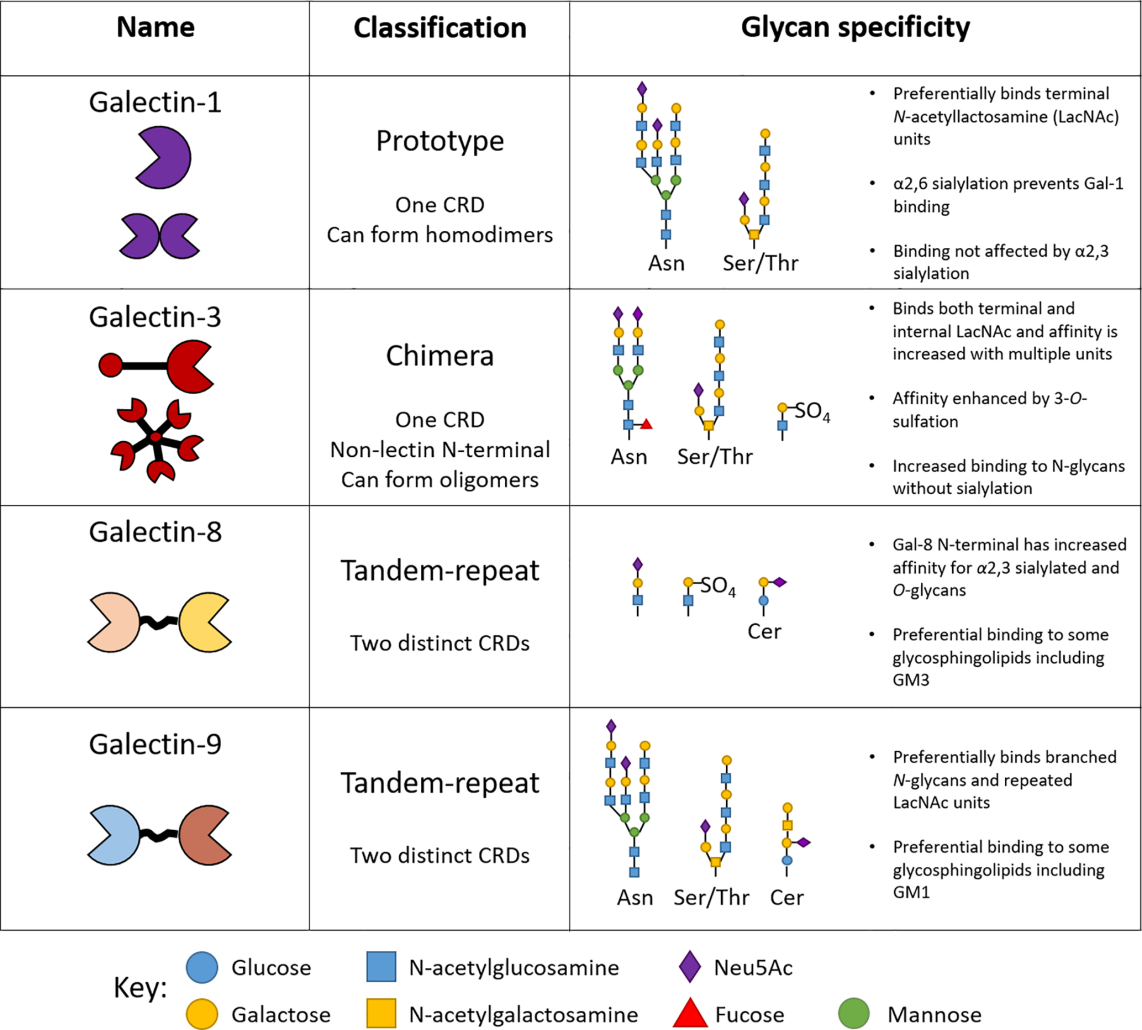
Structure and specificity of human endothelial-expressed β-galactoside binding galectins. The classification of galectins into prototype, tandem-repeat and chimera types is based on the number of carbohydrate recognition domains (CRDs) they contain. Each galectin type reserves high specificity for certain glycan motifs.

Galectins recognize β-galactoside-containing glycan side chains on proteins and lipids *via* their unique carbohydrate recognition domains (CRDs) (15). At present, 15 members of galectins have been identified in mammals, of which 12 are associated with genes found in humans (https://www.ncbi.nlm.nih.gov/genbank/) Galectins-1, -3, -8 and -9 are amongst the most studied members since many of their functions have been closely linked to inflammation and disease (2). Importantly, murine and human galectins share approximately ~79% protein sequence homology (16), allowing their function to be studied in pre-clinical murine models. Galectins can be classified into three distinct groups; prototype, tandem repeat, and chimeric as determined by their structural properties (**Figure 2**). Oligomerization of galectin monomers is typically required for the formation of functional galectin-glycan lattices on the surface of cells (17). Additionally, and despite the absence of a classical secretory signal, galectins are released into the extracellular compartment and mediates extracellular matrix (ECM) assembly and remodeling through binding to ECM components (laminin, fibronectin and vitronectin) (18).

Key conserved amino acids in the CRDs across different galectins are responsible for the specific binding to β-galactoside-containing glycans. Despite these similarities, each galectin type reserves specificity for particular branched glycan’s and glycan motifs attached to proteins and lipids alike (**Figure 2**) (19). For example, the presence of α2,6-linked sialic acid prevents binding of galectin-1 to glycan chains, whilst the same motif enhances the affinity of galectin-8 for its ligand, determining important interactions regulating inflammation (**Figure 2**) (17). The structural determinants of galectin-glycan recognition have recently been reviewed in (20). The effects of glycan modification on galectin–glycan interactions has been reviewed elsewhere and are summarized in **Figure 2** (17, 21). Whether or not structural differences contribute to distinct roles for individual galectin members is yet to be fully elucidated and since galectins are expressed by many cell types it is important to consider both the exogenous and endogenous function of galectins in the context of specific cell types and their physiological interactions.

## Regulation of Galectin Expression in Inflammation

To play a role in leukocyte trafficking, galectins must be present at the site of inflammation where endothelial-leukocyte interactions occur. Expression of galectin-1, -3, -8 and -9 mRNA and protein have been detected in *in vitro* cultured human macro and microvascular ECs, with expression largely intracellular (22). The release of soluble galectin-1 and -8 from human macro- or microvascular ECs respectively, has also been reported (23, 24). Immunohistochemistry (IHC) on sections of human placenta, liver, kidney and colon tissue revealed that low levels of galectin-1 were consistently detected, whilst galectin-3, -8 and -9 expression was detected at variable levels in some but not all of the tissue sections (22). Interestingly, the cellular localization of endothelial galectin expression is highly variable between the different vascular tissue beds (22, 25). Specialized high endothelial venules (HEV) present in sections of human lymph node and tonsil tissue showed higher levels of galectin-9 expression than ECs in other vascular beds (26). Conversely, galectin-3 was not detected and galectin-1 expression was relatively low in HEV from healthy human lymph node sections (25, 27). The variable expression of galectins reported in lymphoid tissues could be indicative of distinct immunoregulatory roles between the galectin types, since the HEV reside within close proximity to leukocytes within the tissue.

An additional consideration when studying ECs is that they possess a complex system of mechanosensitive proteins that respond to hemodynamic forces (28). In response to physiological blood flow, ECs maintain an atheroprotective environment by suppressing gene expression of inflammatory, pro-apoptotic and proliferative pathways *via* mechanotransduction pathways (29). The expression of integrins and adhesion molecules, including PECAM, are modulated *via* this mechanism. Perturbations in shear stress can disturb EC mechanosensitive protein signaling, contributing to the pathophysiological angiogenesis in tumor vasculature where low shear stress is experienced, and driving inflammation in atherosclerosis-prone vascular niches and flow-obstructing pathologies where shear stress is disturbed (30, 31). Deep sequencing of the transcriptome of human umbilical vein EC (HUVEC) and human aortic EC (HAEC) in response to patterns of shear stress has identified changes in expression of galectins not only in response to specific flow patterns, but also across EC type (32). Collectively, these findings suggest that expression of individual galectins is highly dependent on the tissue microenvironment and that a more in-depth comparison of the patterns of galectin expression across different vascular beds could be insightful for understanding galectin regulation and function in this context.

As is the case with many immunoregulatory proteins, galectin expression is modulated with EC activation in response to inflammatory mediators, pathogen exposure, and injury (22, 33–35). A concentration-dependent increase in expression of galectin-9 mRNA and protein was observed *in vitro* following treatment of primary HUVEC with double-stranded RNA viral mimetic, polyinosinic-polycytidylic acid [poly(I:C)], a Toll-like receptor (TLR) 3 ligand (33, 34). Similarly, through the TLR4 pathway, lipopolysaccharide (LPS) stimulation up-regulated surface expression of galectin-8 in human microsvascular EC (HMVEC), suggesting a specific role for galectins in viral and bacterial related infections (24). More basic chemical mediators involved in acute and chronic immune responses have also been shown to differentially regulate endothelial galectin expression. Surface expression of galectin-3 on HUVEC is increased in response to treatment with IL-1β (36). Interestingly, stimulation of HUVEC with IFN-β/γ did not increase expression of galectin-3 (or galectin-1), though a significant increase in galectin-9 surface expression was observed (26). More complex cocktails of soluble disease mediators, such as those released in the conditioned culture medium from colon carcinoma cell lines and in mixtures of oxidized low-density lipoproteins, a known driver of atherosclerosis and cardiovascular disease, stimulated galectin-1 translocation to the cell surface in HUVEC and HAEC (22, 25). The link between increased galectin expression and inflammation is well established, and elevated levels of galectin-3 in the serum and tissue of IMID patients is now recognized as a biomarker for detecting early stages of autoimmune and chronic inflammatory disease (37). Similarly, elevated serum levels of galectin-3 are detected in various cancers and even more so in metastatic disease, correlating with increased levels of metastasis-promoting cytokines released from vascular endothelial cells (38). The galectin expression profile within the tumor vasculature itself highlights a role for endothelial galectins in disease pathogenesis, where soluble factors secreted from tumor cells have been shown to induce galectin-1 expression and translocation to the EC surface to promote tumor angiogenesis and inhibit T-cell migration across the endothelium (22, 23, 39). The link between galectins and tumor angiogenesis, metastasis, and immune suppression could make for an attractive immunotherapy target in combination with currently available cancer therapeutics. Blockade of vascular endothelial growth factor (VEGF) is the current standard of targeted anti-angiogenic therapy and offers variable treatment efficacy dependent on the cancer type (40). Perhaps galectin-1 could be a more appropriate and effective target, even more so if used in combination with targeted therapy such as CAR-T cells to increase tumor infiltration. As has been confirmed with *in vitro* studies, galectin expression in leukocytes is also modulated in response to stimulation with various inflammatory mediators and could be a major influence on the differential expression observed in inflamed tissue and patient serum samples (41). As such, soluble galectins released from immune cells can contribute to the differential levels of galectins observed in inflamed tissue and circulation, potentially counteracting endothelial cell-bound and expressed galectin functions. Distinguishing the specific effects of endothelial galectins on leukocyte trafficking and pathogenesis from the roles of alternative sources of galectins in the inflamed microenvironment is necessary to fully explore the potential for therapeutic intervention.

## Leukocyte Trafficking Regulation by Endothelial Galectins

The interaction between glycans and glycan-binding proteins is crucial at all stages of leukocyte trafficking (1) and remains an active area of research. A large proportion of the literature investigating galectins in inflammation report pro- and anti-inflammatory functions of exogenous galectins. In the absence of inflammatory stimuli, galectin-1 has been reported to promote neutrophil migration *in vitro* (42). Conversely, in the presence of acute inflammatory mediators IL-8 and TNFα, neutrophil chemotaxis was inhibited by galectin-1 (43, 44). Similarly, the transmigration of modified T-lymphocyte cell lines across stimulated HUVEC was significantly inhibited by galectin-1 compared to unstimulated HUVEC (23). Local injection of galectin-3 into the dorsal air pouch was shown to increase monocyte infiltration (45). More recently, galectin-3 was shown to directly dimerize with the chemokine CXCL-12 *via* an interface containing the GAG‐binding motif, inducing anti‐chemokine effects and inhibiting CXCL-12‐induced migration of monocytic THP‐1 cells and neutrophils *in vitro* (46). This latter finding could indicate an endothelial-specific galectin function whereby soluble galectin-3 released from ECs binds to the cell surface to mediate the interaction between leukocytes and CXCL-12. This concept is supported by the study from Yamamoto et al., which reported enhanced binding of T-cells, B-cells, neutrophils, eosinophils and monocytes to HUVEC following pre-incubation with increasing concentrations of both galectin-8 and -9 (47). Interestingly enhanced binding was not observed with galectin-1 or -3, although others have shown that oligomerization of galectin-3 at the EC surface was observed by fluorescence resonance energy transfer (FRET) and facilitated leukocyte clustering at the tricellular corners of HUVEC monolayers where leukocytes preferentially transmigrate (48). Galectin-9 was originally identified as a potent chemoattractant and activator of eosinophils (49). It has since also been shown to bind to protein disulfide isomerases on T-helper 2 cells (Th2), influencing their migration through recombinant galectin-9 coated matrigel by increasing the reduction of disulfide bonds on integrins (50). In the absence of inflammatory stimuli, galectin-9 has been shown to significantly induce monocyte chemotaxis *in vitro* compared to control (51). This finding was also supported *in vivo*, with increased monocyte and macrophage infiltrate in isolated knee tissue from mice receiving galectin-9 *via* intra-articular knee injection (51). The earlier reports strongly suggest a positive role for galectin-9 in driving Th2-type immune responses that could be contributing to the development of diseases such as allergic asthma. However, the substantial increase in galectin-9 expression in ECs following stimulation with Poly : IC and IFNγ, and the increased monocyte and macrophage infiltrate observed in response to galectin-9 injection, suggests that galectin-9 mediated immune responses may be broader than Th2-type only. Together, these findings highlight a potential role for galectins as both pro- and anti-inflammatory molecules affecting leukocyte trafficking dependent on the mediators and cell types in the local microenvironment. It is also worth noting that the glycosylation patterns of leukocyte surface molecules change upon cell activation, and as such, are worth considering when studying the interactions between glycans and glycan-binding proteins to elucidate context specific roles and functions. With only a limited amount of research on the secretion of galectins from ECs, we cannot predict that the results observed in response to exogenous galectins would translate to such function *in vivo*. Thus, further investigation is required to understand the regulators of endothelial galectin secretion and the impact of this on leukocyte migration *in vivo*.

Galectin knockout mice and knock-down studies have been invaluable for studying the endogenous functions of galectins in a more physiological context. Galectin-1 appears to exhibit anti-inflammatory functions since extravasation to inflamed cremaster tissue was enhanced in galectin-1 knockout mice compared to wildtype (44). This mirrored *in vitro* results showing neutrophil recruitment and rolling along TNF-α stimulated HUVEC was enhanced following galectin-1 knockdown in ECs (44). Similarly, endogenous galectin-1 appeared to inhibit T-lymphocyte capture, rolling and adhesion to stimulated HUVEC under physiological flow (52). Interestingly in an acute model of edema, we found that the absence of galectin-1 did not exacerbate the inflammatory response and recruitment of leukocytes as predicted. This in part was due to redundancy amongst galectins, as we demonstrated galectin-9 levels were significantly elevated in these mice and could therefore potentially compensate for the absence of galectin-1 and promote resolution (53). Galectin-3 on the other hand, has been shown to promote leukocyte recruitment *in vivo*, as impaired slow rolling and emigration of leukocytes to IL-1β stimulated cremasteric postcapillary venules of galectin-3 knockout mouse was observed by intravital microscopy (54). Interestingly, these galectin-3 null mouse ECs showed reduced surface expression of ICAM-1 and E-selectin following treatment with IL-1β and vehicle compared to wildtype, suggesting endothelial function might be impaired in the absence of endogenous galectin-3 (54). There is limited research on the roles of galectin-8 and -9 on leukocyte trafficking, especially *in vivo.* The correlation between the results from *in vivo* studies using galectin knockout mouse models and *in vitro* studies using galectin-knockdown ECs support the endogenous galectin function on leukocyte trafficking and inflammation. Despite these convincing reports, an endothelial-specific galectin knockout mouse model is necessary to explore and understand mechanistic pathways and regulators of galectin function in the endothelium. Several endothelial-specific Cre and Cre/ERT2 mouse models have successfully been used in the vascular biology field and should be exploited to study endothelial galectin function.

## Therapeutic Potential of Targeting Endothelial Galectins

The therapeutic potential of targeting galectins for the treatment of inflammatory and autoimmune diseases is currently being explored and showing promising signs of success in early clinical trials (55). Whilst the current and most developed approach is focused on inhibiting galectin-3 with complex carbohydrate mimetics, the potential benefits of administering galectin-1 to mimic its anti-inflammatory effects are also being considered as treatment options in acute myocardial infarction, ischemic stroke and autoimmune diseases (56, 57). Whilst both approaches offer a seemingly promising route to treating chronic inflammation, the systemic approach to treatment might lead to non-specific effects and reduced treatment efficacy. The use of targeted delivery to affected tissues and cells might optimize the potential benefits of drug delivery mimicking or modulating the effects of galectins *in vivo.* Whilst more remains to be understood about the regulation and contribution of endothelial galectins to leukocyte trafficking in acute and chronic inflammation, the interaction between EC-galectins and leukocytes observed *in vivo* and *in vitro* suggests specific pro- and anti- inflammatory, or even pro-resolution roles that could be manipulated for patient benefit.

## Conclusions and Perspectives

Over the last two decades, there has been a steady rise in the number of publications detailing the involvement of galectins in widespread physiological functions, including inflammation, immune responses, apoptosis, autophagy and angiogenesis. Despite a clear link between galectins and disease there still remains ambiguities around the mechanisms by which galectins contribute to pathology, particularly in a cell-specific context since galectin expression and function is diverse and complex. Thus, the challenge remains to gain mechanistic insight, particularly with regards to endothelial galectins in leukocyte trafficking and inflammation to uncover the most appropriate routes for clinical intervention in treatment of IMIDs.

The conditions under which cells and their surface expressed molecules are glycosylated is complex and differs depending on cell state of differentiation, activation and disease (58). As such, attention to the physiological context under which the function of sugar-binding proteins are studied is crucial. A recent study from the Huang group examined the glycosylation-dependent interactions of galectins with intracellular and surface expressed ligands through proximal labeling of cells with galectin-fusion proteins (59). The application of this technique to ECs exposed to inflammatory mediators and/or culture under fluid shear stress could be extremely valuable for understanding the mechanisms and regulators of galectin-glycan interactions in context.

Critically, *in vitro* culture of ECs still remains problematic. The distinct expressional changes between human EC types in response to different shear stress patterns emphasizes the importance of choosing the right models, with consideration for cell type and contextual interactions, to study endothelial-specific responses *in vitro* (32). Whilst many research groups have attempted to mimic physiological flow conditions using expensive and/or specialist equipment, the problem still remains that the complex physiological EC environment cannot be mimicked *ex vivo.* One such example of this is that cultured ECs display deficiencies in the glycocalyx, casting doubts on the reliability of using ECs *in vitro* as a method to study vascular function and role in pathology (60). With major developments in organ on chip technology, we may have greater success in elucidating the role and mechanisms of endothelial galectins in a tissue specific context (61). Finally, the imminent prospect of galectin-targeted therapeutics is encouraging, and whilst interest in galectins remains high and spread across multidisciplinary subjects our understanding of galectin function will only develop further in the coming years.

## Author Contributions

AL, HM, and AI wrote the manuscript. All authors contributed to the article and approved the submitted version.

## Funding

AL is supported by Wellcome Trust Mechanisms of Inflammatory Disease (MIDAS) PhD studentship [222392/Z/21/Z]. HM is supported by the MCR [MR/T028025/1]. AI is supported by Birmingham Fellowship and AMS Springboard Award [SBF003\1156].

## Conflict of Interest

The authors declare that the research was conducted in the absence of any commercial or financial relationships that could be construed as a potential conflict of interest.
